# Macrophages and Foam Cells: Brief Overview of Their Role, Linkage, and Targeting Potential in Atherosclerosis

**DOI:** 10.3390/biomedicines9091221

**Published:** 2021-09-14

**Authors:** Anastasia V. Poznyak, Nikita G. Nikiforov, Antonina V. Starodubova, Tatyana V. Popkova, Alexander N. Orekhov

**Affiliations:** 1 Skolkovo Innovative Center, Institute for Atherosclerosis Research, 121609 Moscow, Russia; 2 Laboratory of Angiopathology, Institute of General Pathology and Pathophysiology, 125315 Moscow, Russia; nikiforov.mipt@googlemail.com; 3 National Medical Research Center of Cardiology, Institute of Experimental Cardiology, 121552 Moscow, Russia; 4 Institute of Gene Biology, 119334 Moscow, Russia; 5 Federal Research Centre for Nutrition, Biotechnology and Food Safety, 2/14 Ustinsky Passage, 109240 Moscow, Russia; avs.ion@yandex.ru; 6 Medical Faculty, Pirogov Russian National Research Medical University, 1 Ostrovitianov Street, 117997 Moscow, Russia; 7 V.A. Nasonova Institute of Rheumatology, 34A Kashirskoye Shosse, 115522 Moscow, Russia; popkovatv@mail.ru

**Keywords:** atherosclerosis, inflammation, macrophage, foam cell

## Abstract

Atherosclerosis is still one of the main causes of death around the globe. This condition leads to various life-threatening cardiovascular complications. However, no effective preventive measures are known apart from lifestyle corrections, and no cure has been developed. Despite numerous studies in the field of atherogenesis, there are still huge gaps in already poor understanding of mechanisms that underlie the disease. Inflammation and lipid metabolism violations are undoubtedly the key players, but many other factors, such as oxidative stress, endothelial dysfunction, contribute to the pathogenesis of atherosclerosis. This overview is focusing on the role of macrophages in atherogenesis, which are at the same time a part of the inflammatory response, and also tightly linked to the foam cell formation, thus taking part in both crucial for atherogenesis processes. Being essentially involved in atherosclerosis development, macrophages and foam cells have attracted attention as a promising target for therapeutic approaches.

## 1. Atherosclerosis

Atherosclerosis implies the concentration of fatty and/or fibrous material within the intima. The definition of “atherosclerosis” originated from the Greek language and means “gruel” or “porridge” which literally illustrates the lipid material appearance that exists in the nucleus of a typical atherosclerotic plaque (or atheroma) [1]. After a while, the atherosclerotic plaque can become more fibrous and store calcium minerals [2]. Progressive atheroma is able to penetrate the lumen of the artery, which consequently disrupts the bloodstream and leads to tissue ischemia. At the same time, those atheromas that do not create a flow-limiting obstruction can destroy and cause a blood clot formation that may clog the lumen, which, as a result, can lead to more acute ischemia [3]. It is known that atherosclerotic cardiovascular diseases (CVD) are the main cause of vascular diseases spread globally. Affecting the heart’s own blood circulation can cause acute coronary syndromes: for example, a myocardial infarction or stable angina pectoris [4].

Ischemic strokes and transient cerebral ischemic attacks can occur as a consequence of atherosclerosis [5]. As a result, aneurysms may form, which can also form in the abdominal aorta. If the peripheral artery is affected, episodic lameness, ulceration, and gangrene may appear, which potentially threaten the viability of the limbs. As before, atherosclerosis remains the leading cause of death worldwide [6].

Despite the improvement of preventive measures, numerous patients still die from acute complications of atherosclerosis outside the hospital. However, when a patient with an acute atherosclerosis manifestation seeks help promptly, modern treatment strategies in most cases save lives [7]. Such progress in cardiovascular medicine is an important and remarkable example of how the clinical application of scientific discoveries can benefit patients. Regardless of the successes, there is still a lot of work to be done to apply more effectively and fairly what we already know in practice [8]. It is necessary to set a higher bar for ourselves to reduce the burden of residual risk, which, alas, is still quite high. Quite a large number of people experience acute coronary syndromes, however, this does not exclude such a consequence as a violation of cardiac function, which is the basis for heart failure [9].

## 2. Inflammatory Role of Monocytes

The first front line of body defense from pathogenic microorganisms or tissue harm is monocytes. Circulating monocytes comprise three subsets: classical CD14++CD16− monocytes, nonclassical CD14 + CD16++ and intermediate CD14 + CD16+ [10]. Monocytes differentiate into macrophages immediately after they are at the site of danger due to chemotaxis. Together with certain macrophage-residents of tissues that maintain their pool without the active participation of blood monocytes, macrophages derived from monocytes are involved in the inflammatory process. As a response to specifically occurring molecular patterns linked with pathogens and damage (PAMPs (pathogen-associated molecular patterns) and DAMPs (damage-associated molecular pattern), and host inherited signaling molecules, macrophages adopt a variety of functional phenotypes [11]. There are two opposite phenotypic states which are represented by the classical model of macrophage polarization: (1) the “classic” proinflammatory macrophage M1, generated by bacterial lipopolysaccharide (LPS) and/or gamma interferon (IFN-ɣ), (2) and the “alternative” anti-inflammatory macrophage M2, which may be stimulated by interleukin 4 (IL4) [12]. Nevertheless, recent development in functional characterization shows that macrophage phenotypes are not restricted to the extremes of M1 and M2, but more likely are a spectrum of phenotypes linked with differential cytokine development and functional parameters. This functional plasticity of macrophages is regulated by transcriptional reprogramming, which is reached by changing the availability of chromatin and the epigenetic landscape [13]. In the case of a disease, chronic inflammation is able to result in reverse remodeling of macrophage reactions and provoke a change in their phenotypes, which can lead to proinflammatory macrophages’ growth in diabetes or anti-inflammatory macrophages’ growth in cancer [14]. The basis of the pathogenesis of the disease in atherosclerosis is the dysregulation of macrophages. The generation of atypical states of macrophage activation, which include the characteristics of pro- and anti-inflammatory phenotypes, is due to the action of modified lipids, cholesterol crystals, and mediators on monocytes [15].

These changes are linked to transcriptional and epigenetic reprogramming and are modulated by transcription factors and epigenetic. The awareness of the basic regulatory mechanisms can make a contribution to the invention of the new treatment, e.g., by blocking an unnecessary pathway or reprogramming macrophages to a more suitable phenotype [12].

## 3. Inflammatory Role of M1 and M2 Macrophages

The polarization system of T-cells is found on the transcriptome, phenotype, and functions and is well-proven [16]. Along with this system, the affected macrophages are strongly impacted by microenvironment signals and are polarized into multiple classes with various phenotypes and functions. Because of the macrophages’ unsteadiness during the isolation process and the dissimilarities in animal and human phenotype models, accurate studies have become limited [17].

In a simpler dichotomy, immune-activated proinflammatory macrophages (M1) and immunomodulating alternatively triggered macrophages (M2) are the most traditional classification that illustrates 2 types of T helper cells—Th1 and Th2 [13]. This classification shows extreme phenotypes of complex activation states. As a rule, M1 macrophages are polarized by Th1 cytokines (for example, interferon (IFN-γ) and TNF) and molecular complexes linked with pathogens (PAMPs) consisting of lipopolysaccharides and lipoproteins [18]. Through the regulatory interferon factor 5 (IRF5), the granulocyte-macrophage colony-stimulating factor (GM-CSF) participates in the inflammatory process. M1 macrophages develop increased levels of proinflammatory cytokines (which are IL-6, IL-12, IL-23, TNF-α, and IL-1β) and chemokines associated with Th1 recruitment (CXCL-9, CXCL-10, and CXCL-11). In addition, levels of produced IL-10 are decreased. Regardless chronic activation, M1 macrophages are also able to trigger the NADPH oxidase system and, as result, produce reactive oxygen species (ROS) and nitric oxide (NO), leading to chronic tissue detriment and wound curing worsening [19].

At this stage, M2 macrophages play an important role in balancing the proinflammatory response, the function of modulating inflammation, eliminating apoptotic cells, boosting the process of formation of new blood vessels (angiogenesis) and scar formation (fibrosis), and stimulating tissue recovery. Meanwhile, M2 macrophages are usually triggered in response to Th2-related cytokines, including IL-4, IL-33, and IL-13 [20]. Activated M2 macrophages have immunomodulatory properties and have decreased levels of IL-12 along with increased levels of anti-inflammatory cytokines (IL-10 and TGF-β), as well as chemokines (CCL17, CCL22, and CCL24). Thus, taking into account the distinction between activation signals and gene expression profiles, M2 macrophages can be alternatively separated into four subgroups [21,22]:

(1) M2a—M2a macrophages are induced by IL4 and IL 13 and generate increased levels of CD206 and IL-1 receptor antagonist [23];
(2) M2b—M2b macrophages are extraordinary and trigger immune complexes, IL1β and PAMPs, and produce both the proinflammatory cytokines (IL-1, IL-6, and TNF-α) and the anti-inflammatory cytokine IL-10 [24];
(3) M2c—the most prominent anti-inflammatory subtype triggered by IL10, TGFß, and glucocorticoids and produce IL10, TGFß and pentraxin 3 (PTX3) [25];
(4) M2d—are triggered by diphtheria toxin receptor (DTR) signals and have angiogenic properties that play a role in both plaque growing and tumor development [26].

The key signal for M2 polarization is the activation of the γ receptor, which is triggered by the peroxisome proliferator (PPAR-γ), and the signal converter and a trigger of transcription pathways 6 (STAT6) [22,27]. Both M1 macrophages and M2 macrophages are staying in different areas of the plaque. Staining with M1 macrophage markers is mainly limited to the shoulder of plaques subject to break, one of the most unbalanced areas inside the plaque [13]. Meanwhile, M2 macrophages markers are usually present in the adventitia of vessels or the stable plaques areas. It is worth noting that M1 macrophages are also more common in lesions in patients with heart attack and coronary heart disease than M2 macrophages [18,22].

## 4. Inflammatory Role of Macrophages of Other Phenotypes

**Figure 1:**
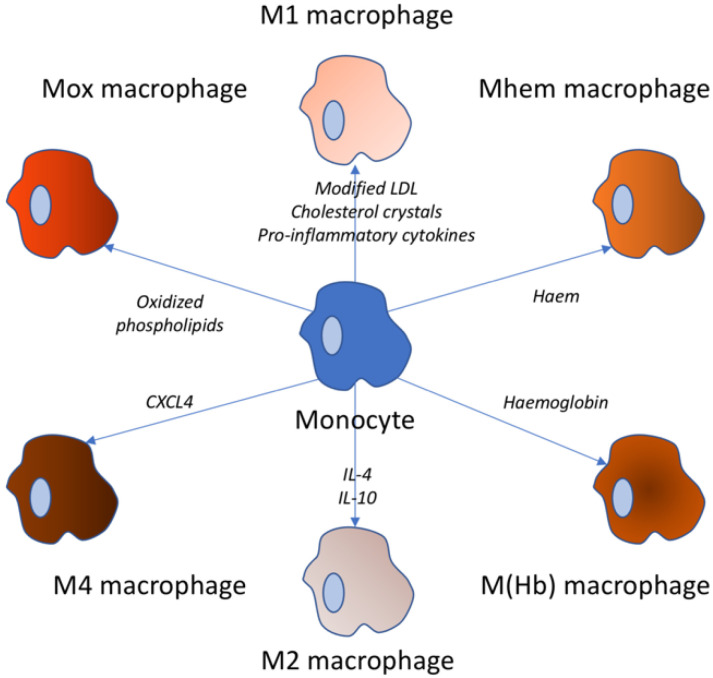
Variety of macrophage types. These types of macrophages can be observed in atherosclerosis. The stimuli triggering the respective transformation from the monocyte are noted near the arrow.

Along with a comprehension of the phenotypes and functions of affected macrophages, it was proved that the M1-M2 dichotomy ultimately does not demonstrate complex subsets of macrophages in atherosclerosis, which are highlighted in Figure 1. Stimuli change spatiotemporally and bring macrophages to a wide range of activation states, rather than to a stable analogous polarization, which may obstruct phenotype stability maintenance of isolated macrophages. A new method of macrophage classification is by stimuli: M (IFN-γ), M (IL-4), and M (IL-10) [13,28].

Not so long ago, Piccolo et al. triggered macrophages by double stimulation of IFN-γ and IL-4, which are suppressors of macrophages of the M1 and M2 phenotype, and established that co-stimulation with two contrary stimuli led macrophages to an intermediate state, which we are able to entitle M (IFN-γ-IL-4), and showed both transcriptomes of specific genes of the M1 and M2 type [29].

Apart from M1–M2, oxidized phospholipids are able to trigger the Mox phenotype in macrophages by activating the Nrf2 transcription factor in mouse models. In progressive plaque, Mox macrophages are nearly 30% of the aggregate number of macrophages [30]. These cells express proinflammatory markers, such as IL1β and cyclooxygenase 2, and show deficient phagocytic and chemotactic abilities. The destruction of microvessels in the lesion site lets out red blood cells, which are able to be phagocytized by macrophages, and then trigger them into the M(Hb) and Mhem phenotypes [31].

Macrophages of M(Hb) subtype can form hemoglobin-haptoglobin complexes in vitro and represent the CD206 + CD163 + phenotype. Macrophages M(Hb) are characterized by high activity of liver receptor X a (LXRa), which results in an elevation in cholesterol outflow and a decrease in lipid accumulation, as well as elevation in ferroportin expression, which, in turn, led to an intracellular iron storage decrease and greater secretion of anti-inflammatory factors (such as IL-10) [32]. The Mhem phenotype is polarized by heme and is characterized by cyclic AMP high expression—dependent transcription factor—(ATF-) 1 and heme oxygenase 1 (HO1) and inhibited oxidative stress or lipid storage, analogous in properties to M(Hb) macrophages [33].

M(Hb) cells and Heme cells are referred to phenotypes linked with hemorrhage. They are usually persistent to transformation into foam cells, inhibiting oxidative stress and potentially performing atheroprotective functions [34]. Chemokine 4 of the CXC motif (CXCL4) chemokine triggers macrophages of the M4 phenotype in human atherosclerotic plaques. In turn, M4 macrophage phenotype is CD163. It is differed by the expression of both MP-7 and the calcium-binding protein S100A8 and, in addition, the manifestation of proinflammatory and proatherogenic properties [35,36]. Remarkably, the M1, M2 phenotypes and bleeding-related phenotypes can shift with each other while the M4 macrophage phenotype appears to be permanent.

## 5. Macrophages in Atherosclerosis

During atherosclerosis development, five to eight macrophages in the mouse aorta, obtained mainly from circulating monocytes and local proliferation, grow up to 20-fold. There is proof that vascular smooth muscle cells are able to differentiate into a macrophage-like state [37]. In humans, the set of monocytes in the intimal space arise at a very early age. The initial stages are manifested in infants under the age of one year, and atherosclerotic plaques are common in teenagers and young adults [38]. Thus, atherosclerotic cardiovascular disease (ASCVD) is a chronic inflammatory process that develops during the life and, unfortunately, for many ends with a serious unfavorable and sometimes fatal outcome [39].

One of the important features of ASCVD is the ingress of lipoproteins in the body and their storage by arterial macrophages, which results in foam cells formation. Due to the accumulation of foam cells lipids also accumulate in the plaque, which contributes to the steady growth of plaque. Macrophages promote the upkeep of a local inflammatory response by releasing proinflammatory cytokines, chemokines and developing reactive oxygen and nitrogen forms [40]. Macrophages also interact with vascular smooth muscle cells, enhancing the inflammatory cycle by developing additional proinflammatory cytokines and extracellular matrix components, which additionally contribute to the retention of lipoproteins. The plaque macrophage’s capability to migrate is low, so they prevent the resolution of inflammation and contribute to the lesions’ development into complex, tear-prone plaques [41]. In addition, stable inflammation stimulates apoptosis of macrophages and, in the absence of effective efferocytosis, results in ruins and apoptotic cell storage, contributing to the establishment of a necrotic nucleus in an atherosclerotic plaque [42].

Plasticity is the defining distinguishing feature of macrophages. Plasticity allows macrophages to develop an individual response to local stimuli of the microenvironment. During the inflammatory process, macrophages can either stimulate inflammation or vice versa eliminate it during the recovery of wounds and tissues. The traditional model of macrophage activation describes both pro- and anti-inflammatory macrophages with different physiological purposes and triggers. At the widest possible level, macrophages should be categorized either as (1) M1-classically activated; or as (2) M2-alternatively activated [43,44].

In vitro, M1 macrophages are polarized in response to toll-like receptor ligands, interferons, molecular complexes linked with pathogens, lipopolysaccharides and lipoproteins fed by glycolysis, M1 macrophages stimulate tissue destruction and secrete proinflammatory factors, including increased IL (interleukin)-1β, IL-6 and TNF-α (tumor necrosis factor-α) levels [45]. Pursuing their inflammatory phenotype, they express proinflammatory transcription factors as nuclear factor-kB and STAT (signal transformer and transcription activator)-1. M2 macrophages exist at the other spectrums’ end with a phenotype hinged on the oxidation of the fatty acids, and anti-inflammatory properties [46]. M2 macrophages are polarized in response to the cytokines IL-4 and IL-13 and secrete anti-inflammatory factors including collagen and IL-1 receptor agonist, IL-10. M2 macrophages are characterized by the expression of CD163 (cluster of differentiation 163), mannose receptor 1, resistin-like β, and an increased level of arginase-1 [47].

Within the framework of plaques, macrophages belonging to traditionally included and alternatively activated subsets participate in both human and mouse lesions. Notably, the predominant subtype is M1 [48]. In human lesions, macrophages expressing proinflammatory markers are located in unstable areas predisposed to break, and M2-like macrophages are located in persistent areas and adventitia. However, recent data demonstrate that macrophages exist in the activation continuum, and that the M1/M2 classification system is a very strong simplification of the heterogeneity of macrophages and their various functions [49].

Several classifications are described in the context of murine ASCVD [37]. These alternative phenotypes involve Mhem macrophages, which are contained in hemorrhages and which phagocytize, as well as use red blood cell residues and hemoglobin deposits [25]. This subset is atheroprotective and resistant to the development of foam cells. It is shown with their increased expression of cholesterol transporters ABCA1 (ATP-binding cassette transporter A1) and ABCG1 (ATP-binding cassette transporter G1) and nuclear receptors, LXR-α and LXR-β [50]. Mox macrophages (proatherogenic subset) are triggered by modified phospholipids and defend against oxidative stress utilizing a 2-nuclear factor-related factor 2 linked with erythroid, mediated by the expression of the antioxidant enzymes such as heme oxygenase 1, thioredoxin reductase 1, and sulfiredoxin–1 [51,52]. It is reported that in mice with hypercholesterolemia, Mox macrophages make up 30% of plaque macrophages, with subsets M1 equals 40% and M2 equals 20% of the remaining cohort, accordingly [53]. Ultimately, M4 macrophages are a subset polarized by platelet factor 4. This population is found in human lesions that have increased expression of matrix metalloprotease 7 and S100A8. M4 macrophages are defined as atherogenic based on their development of proinflammatory cytokines (IL-6 and TNF-α) and defective phagocytic properties [13,54]. For the first time, the heterogeneity of macrophages in plaques was evaluated using immunohistochemistry and, at the molecular level, using laser capture microdissection. In response to technological advances, which also involve mass time-of-flight cytometry and single-cell RNA sequencing, it has become possible to further expand our knowledge about the heterogeneity of macrophages in progressive plaques. As a result, these technologies helped to characterize the heterogeneous nature of plaque macrophages and revealed a fresh, undetected earlier subset of identified ones [55].

Called the activated receptor expressed on myeloid cell macrophages 2 (TREM)^hi^, this subset expresses increased Trem2, Cd9, Ctsd, and Spp 1 genes levels and reduced expression of inflammatory cytokines with attributed biological functions of lipid metabolism and cholesterol outflow [56]. It is assumed that this extraordinary population is fulfilled with cholesterol and represents foam macrophages. Thus, the macrophages of the TREM)^hi^ give an alternative hypothesis, which implies that subsets of macrophages are inflammatory in some places [57].

There are multiple models of macrophage activation. Taken together, they show that macrophages in plaques are able to have solely fractional similarity with the phenotypes of M1 and M2 macrophages [43]. In order to discover the gene expression profiles and transcription pathways that are the basis for the identity and diversity of macrophages in ASCVD, further research is needed. Moreover, it is also important to determine whether the results in mice can be translated into human plaques that have clear phenotypic differences (such as hemorrhage and rupture), in order to develop treatments aimed at reducing the risk of residual inflammation associated with macrophages [58].

## 6. Foam Cells

Following the joining of the ECs, monocytes get through the ECs into the subendothelial area and persist there due to the reduced aptitude to migrate, preventing the resolution of inflammation. Moved by pro-differentiation factors (e.g., macrophage colony-stimulating factor (M-CSF)) monocytes evoke phenotypes similar to macrophages or dendritic cells (DC) [59]. DC are intensively involved in lipoprotein particle purification and transformation into foam cells, which are cytoplasmic and membrane-bound splashes resulting in greater storage of modified LDL in the subendothelial space [4,41]. For this implementation course, several mechanisms have been offered. Some studies have shown that scavenger receptors expressed on macrophages (mainly the scavenger receptor type A (SR-A) and a part of the CD36 family type B) are key markers on affected macrophages that turn into foam cells [60,61]. Obstructed sera trigger the absorption of lipids and the foam cells generation, thus further blocking the local proliferation of macrophages in the lesion [62]. It is worth noting that in the triple knockout models of Apoe−/− CD36−/− Msr1−/− mice, there was no reduction in the foam cells transformation in contrast to Apoe−/− mice, which determines that additional mechanisms for monitoring this process have yet to be specified [63]. Lately, newer scavenger receptors have been established, such as protein 1 associated with the LDL receptor (LRP1) and the lectin-like VLDL receptor 1 (LOX1), which also promote the absorption of lipids. It has been proven that blocking LRP1 in affected macrophages reduces the cholesterol concentration in macrophages [64]. On the contrary, the liver X-receptor (LXR) enabled by oxLDL contributes to the outflow of cholesterol and lowers the expression of proinflammatory factors in macrophages, therefore having a beneficial influence on atherosclerosis [15]. In addition to modified LDL, it was established that the transformation of foam elements is also able to occur due to the receipt of native LDL, independent of receptors. This process is called fluid-phase endocytosis and is based on the activation of phorbol 12-myristate 13-acetate (PMA), a trigger of protein kinase C (PKC) [65].

## 7. Foam Cells Are Not Always Macrophage-Derived

**Figure 2:**
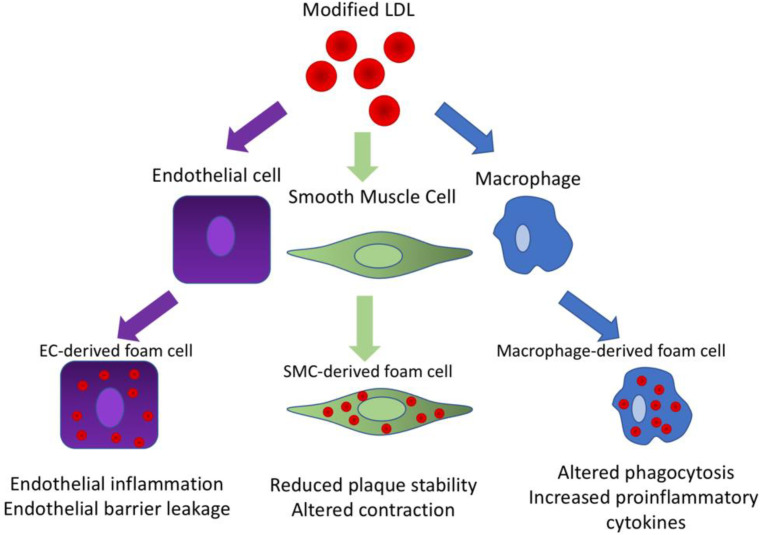
Modified LDL triggers foam cell formation from different cell types.

The classical conception of the formation of atherosclerotic lesions focuses on the generation of foam cells from macrophages derived from monocytes, but a significant part of the foam cells actually originate from the cells of the intimal smooth muscle cells (SMCs), and, moreover, from endothelial cells (see Figure 2) [37]. It was described that under the influence of platelet-derived growth factor β (PDGF-β), SMCs are able to lose their contractile phenotype and transform into a more synthetic phenotype that produces an extracellular matrix and has a regenerating, wound-healing function, which restores and stabilizes the artery wall; and in the atherosclerotic lesions, thickens and stabilizes the fibrous membrane [66]. However, during lesion development, synthetic SMCs are one of the first cell types that remain lipoprotein contents.

Further impact to signals inside the plaque (e.g., TGF-β, oxidized lipids, and cytokines) are able to cause transdifferentiation of synthetic SMCs into foam cells. It is worth noting that cholesterol itself has been shown to trigger transdifferentiation of mouse SMCs and an elevation in the expression of CD68, Mac-2, and ABCA1 foam cell markers [67]. It is also believed that up to 50% of all foam cells in the field of human lesions have SMC- origin. This quantitative assessment is based on co-staining with markers specific to SMC and foam cells, which does not take into account the complete phenotype and the vector of transdifferentiation and may result in a re-evaluation of transdifferentiating cells [68]. However, mouse-based line-tracking tests have allowed us to identify and characterize these transitions in vivo and the underlying epigenetic mechanisms. For example, thus, the Kruppel-Like Factor 4 (KLF4) TF program was identified as an SMC transdifferentiation driver. Myeloid DTF Sp1 (PU 1) has been shown to bind to the KLF4 promoter in response to PDF-β signaling. Like STAT6, KLF4 triggers the anti-inflammatory state of macrophages by inducing a protein triggered by MCP-1 (encoded by the ZC3H12A gene), which inhibits the function of NF-kB and activates the C/EBPb and PPAR-ɣ programs [12].

Although the foam cells obtained from SMC acquire macrophage markers (for example, CD68 and Lgals3), as well as storing cholesterol and lipoproteins, they do not acquire phagocytic or efferocytic abilities and, accordingly, do not turn into real macrophages [53]. However, the simultaneous loss of SMC markers complicates the separation of foam cells obtained from SMS from their analogues obtained from monocytes. In macrophages obtained from monocytes, activation of the KLF4 program results in atheroprotective, anti-inflammatory phenotype [69]. Epigenetic expression of KLF4 is controlled by methylation of its promoter DNMT1. In inflammatory macrophages, KLF4 is hypermethylated, which results in the inhibition of its TF program. While KLF4 plays a neuroprotective role in macrophages, KLF4 in SMCS appears to be proatherogenic, and its loss results in the reduction of the lesion size and an elevation in the stability of plaques. In fact, in cultured SMCS, cholesterol-induced KLF4 expression resulted in KLF4-dependent activation of proinflammatory cytokines, thereby contributing to the formation of a proinflammatory atherogenic plaque [70].

## 8. Therapeutic Strategies Targeting Macrophages

Antiatherosclerotic biomarkers or lipid modulation strategies are nonspecific measures that inhibit macrophage functions and other cells in the plaque (for example smooth muscle cells (SMCs) and endothelial cells (ECs)). Nevertheless, treatment methods that expressly and peculiarly target macrophages are insufficient, and up to now the research has been a preclinical work, possibly due to the complex phenotypic and functional heterogeneity of the affected macrophages [71].

Now, new systems of drug supply (such as NPs, stents, liposomes, glucon shell microparticles, oligopeptide complexes, and monoclonal antibodies) allow the selective modification of macrophages. Macrophage surface markers (F4/80, CD11b, CD68, CD 206) and scavenger receptors enforce distinctive targets for all macrophages or different subsets [72]. In combination with surface coating receptors or depending on their chemical properties, these systems are able to provide drugs or RNA interference (RNAi) to local atherosclerotic plaques or specific subsets of macrophages, making changes with minimal nontargeted effects and toxicity [73]. Targeting macrophages, various approaches are able to be used to modulate their activity, including impelling cell apoptosis, cell proliferation suppression, and administering anti-inflammatory drugs.

Verheye et al. established that the rapamycin inhibitor everolimus, transferred to plaques in a rabbit found on a stent, resulted in autophagy in macrophages without influencing the number of SMCs [74]. In a mouse model with a high-fat diet, clodronate liposome injection effectively exhausts the microphone of visceral adipose tissue and blocks weight gain and metabolic disorders caused by a high-fat diet. Stoneman et al. found the effect of general ablation directed at macrophages and blood monocytes by creating a transgenic mouse model with the CD11b-DTR by introducing DT [26]. When DT was used at the beginning of atherogenesis, the plaques were significantly reduced. At the same time, the detected plaques were not touched by DT, even though macrophages were lowered to the same level, which demonstrates that the atherogenesis process is more susceptible to a decrease in monocytes/macrophages than stable plaques. Sadly, despite the high-potential results, all the proof has been obtained in vitro or animal models, and further research is required for the development of new drugs and clinical translation.

An alternative option for removing macrophages that affect the polarization of macrophages into an anti-inflammatory phenotype involves not the M1 macrophages, but those of M2 phenotype. Possible targets may be many factors affecting the M2 polarization signals [75]. For instance, it is assumed that dipeptidyl peptidase (DPP) inhibitors, such as gliptins and sitagliptin, can stimulate M2 polarization in vitro by transmitting SDF-1/CXCR4 signals. Thiazolidinediones or TZDs (rosiglitazone and pioglitazone, activators of PPAR-γ), are able to contribute to the polarization of monocytes into the M2 phenotype by changing the expression of M2 markers, such as the mannose receptor (MR) and CD163 [35].

## 9. Therapeutic Strategies Targeting Foam Cells

One of the most significant events in foam cell formation is cholesterol efflux. This process is mediated by ATP-binding cassette transporters ABCA1, ABCG1, as well as SR-B1, the function of which is to maintain cholesterol and phospholipid homeostasis in the cell [15]. The treatment of LDLR−/− mice with PPARα and PPARγ agonists was shown to increase the production of ABCG1 and ABCA1, which contributed to the inhibition of atherosclerosis progression [76]. However, there are other cholesterol-transporting mechanisms, so this approach is limited.

Another approach targeting foam cells is to slow the foam cell degradation, which can potentially have an atheroprotective effect [77]. Among such approaches are promoting efferocytosis of apoptotic macrophages by using LXR ligands or glucocorticoids, activating PPARγ pathways, knockdown of apoptosis inhibitor of macrophages, targeting apoptotic pathways, such as the genetic inhibition of BAX or Bcl-2 and other atherogenic proteins, targeting secondary necrosis pathways (e.g., the clearance of apoptotic cells).

Although, most of the therapeutic strategies involving foam cells were based on the hypothesis of only macrophage origin of foam cells, while today we know the heterogeneity of this kind of cells [78].

## 10. Conclusions

The wide involvement in atherogenesis makes macrophages an interesting target for potential therapeutic strategies. Various approaches can be used for macrophage activity modulation, such as impelling cell apoptosis, cell proliferation suppression, and administering anti-inflammatory drugs. However, treatment strategies that directly target macrophages are insufficient and may be more efficient in complexity with other measures. The same can be said about foam cells. It was long believed that macrophages are their only source, and this misconception severely limited understanding of the therapeutic potential of foam cells. However, over time, more and more new data on the heterogeneity of foam cells appear, which will allow more efficient targeting options for atherosclerosis treatment.

## Data Availability

Not applicable.
